# Characterization of a porcine model of atrial arrhythmogenicity in the context of ischaemic heart failure

**DOI:** 10.1371/journal.pone.0232374

**Published:** 2020-05-04

**Authors:** Sebastian Clauss, Dominik Schüttler, Christina Bleyer, Julia Vlcek, Mehdi Shakarami, Philipp Tomsits, Sarah Schneider, Florian Maderspacher, Kavi Chataut, Anna Trebo, Christine Wang, Jan Kleeberger, Ruibing Xia, Elisabeth Baloch, Bianca Hildebrand, Steffen Massberg, Reza Wakili, Stefan Kääb

**Affiliations:** 1 Department of Medicine I, University Hospital Munich, Campus Großhadern, Ludwig-Maximilians University Munich (LMU), Munich, Germany; 2 DZHK (German Centre for Cardiovascular Research), Partner Site Munich, Munich Heart Alliance (MHA), Munich, Germany; 3 Walter Brendel Centre of Experimental Medicine, Ludwig-Maximilians University Munich (LMU), Munich, Germany; 4 Universitätsklinikum Essen, Westdeutsches Herz- und Gefäßzentrum Essen, Essen, Germany; Scuola Superiore Sant’Anna, ITALY

## Abstract

Atrial fibrillation (AF) is a major healthcare challenge contributing to high morbidity and mortality. Treatment options are still limited, mainly due to insufficient understanding of the underlying pathophysiology. Further research and the development of reliable animal models resembling the human disease phenotype is therefore necessary to develop novel, innovative and ideally causal therapies. Since ischaemic heart failure (IHF) is a major cause for AF in patients we investigated AF in the context of IHF in a close-to-human porcine ischaemia-reperfusion model. Myocardial infarction (AMI) was induced in propofol/fentanyl/midazolam-anaesthetized pigs by occluding the left anterior descending artery for 90 minutes to model ischaemia with reperfusion. After 30 days ejection fraction (EF) was significantly reduced and haemodynamic parameters (pulmonary capillary wedge pressure (PCWP), right atrial pressure (RAP), left ventricular enddiastolic pressure (LVEDP)) were significantly elevated compared to age/weight matched control pigs without AMI, demonstrating an IHF phenotype. Electrophysiological properties (sinus node recovery time (SNRT), atrial/AV nodal refractory periods (AERP, AVERP)) did not differ between groups. Atrial burst pacing at 1200 bpm, however, revealed a significantly higher inducibility of atrial arrhythmia episodes including AF in IHF pigs (3/15 vs. 10/16, p = 0.029). Histological analysis showed pronounced left atrial and left ventricular fibrosis demonstrating a structural substrate underlying the increased arrhythmogenicity. Consequently, selective ventricular infarction via LAD occlusion causes haemodynamic alterations inducing structural atrial remodeling which results in increased atrial fibrosis as the arrhythmogenic atrial substrate in pigs with IHF.

## Introduction

Atrial fibrillation (AF) is the most common sustained arrhythmia and has shown an increase in incidence, prevalence and AF-associated mortality over the last decades [1]. It is an independent risk factor for all-cause mortality and contributes to substantial cardiovascular morbidity, above all stroke and heart failure [2]. Various independent risk factors for the development of atrial fibrillation have been identified so far including valvular heart disease, congestive heart failure, age, diabetes and hypertension. Despite major progress in understanding the underlying pathophysiological mechanisms, therapeutic options with antiarrhythmic drugs or catheter ablations still have limitations and their impact on efficacy and prognosis remains unclear though recent studies found survival benefits [3, 4]. Different mechanism, so-called remodeling processes contribute solitary or combined to the development of AF: (I) electrical remodeling which includes changes in calcium handling, ion currents or electrical conduction, (II) structural remodeling including atrial enlargement and fibrosis and (III) autonomic remodeling with changes in autonomic nerve density and innervation patterns [3]. Since these alterations create a vulnerable atrial substrate any trigger such as a premature atrial capture beat can initiate AF [5]. Although the likelihood of AF maintenance is higher in patients with dilated, i.e. structurally remodeled atria, AF can also occur in structurally healthy atria demonstrating the heterogeneity of the disease [6].

Thus, modeling AF in animals remains challenging and currently no ideal AF model is available. Given that translation of basic research findings into clinical practice is the most important step which limits the development of novel treatment strategies, clinically relevant, close-to-human large animal models are urgently needed. Pigs could serve as valuable model species especially since their anatomy, haemodynamics or electrophysiology are similar to humans [7]. The most commonly used porcine AF model is the tachypacing model which mimics atrial tachycardia as trigger to initiate proarrhythmic remodeling but also causes tachymyopathy. This, however, is rare in humans, whereas the most common cause for AF in patients is myocardial ischaemia. Between 6 and 21% of the patients develop AF after myocardial infarction with AF being associated with poor prognosis in these patients [8–10]. Thus, a pig model for AF in the context of ischaemic heart disease that resembles remodeling processes similar to those found in many patients might be highly relevant: It could be used as suitable model for translational studies and might therefore help to improve patient care in the future. Here we investigated a pig model which reliably mimics the human phenotype of ischaemic heart failure 30 days after an ischaemia-reperfusion injury causing distinct atrial structural remodeling processes and leading to a proarrhythmogenic atrial substrate with subsequent increased atrial arrhythmogenicity. This model can be induced easily and is reproducible which makes it a suitable large animal model to study pathomechanisms underlying atrial arrhythmogenesis after AMI and to translate basic science findings into clinical practice e.g. by testing novel drug compounds or interventional approaches.

## Materials and methods

### Animal instrumentation and ethics

**Fig 1:**
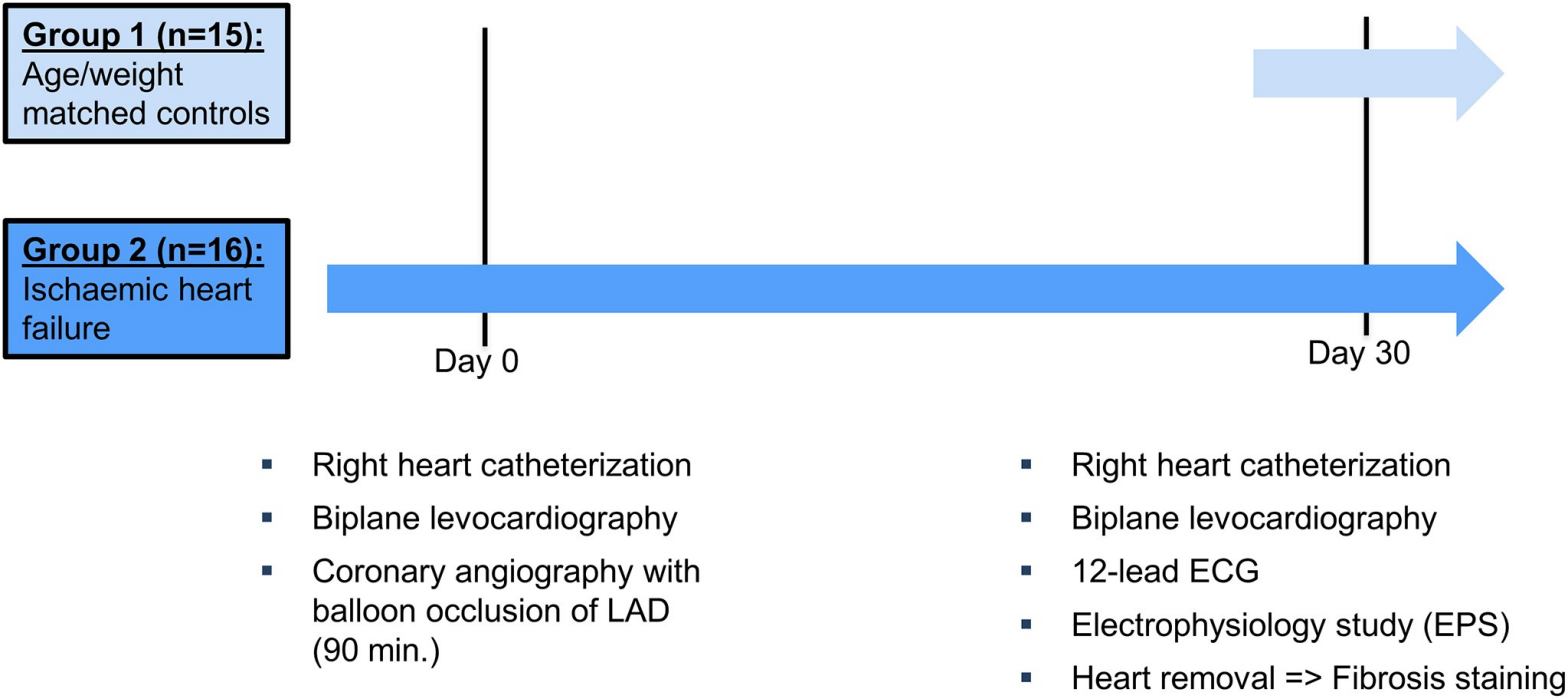
Study protocol. 2 Groups of pigs were studied: control pigs (n = 15) and pigs with ischaemic heart failure (IHF, n = 16). Illustration of experiments performed on day 0 and day 30 in both groups.

All experiments were approved by the *Regierung von Oberbayern* (55.2-1-54-2631-130-10, ROB-55.2-2532.Vet_03-15-73 and ROB-55.2-2532.Vet_02-15-209) and were conducted at the Walter Brendel Centre of Experimental Medicine. All investigations followed the “Guide for the Care and Use of Laboratory Animals” (National Institute of Health, 8^th^ Edition 2011). The study was carried out in German Landrace pigs (3–4 months old, 50–70 kg body weight) obtained from *Landwirtschaftliche Forschungsstation Thalhausen*, Technical University of Munich, Kranzberg, Germany, *Moorversuchsgut*, Ludwig-Maximilians-University Munich, Oberschleissheim, Germany, and *Lehr- und Versuchsgut der LMU*, Ludwig-Maximilians-University Munich, Oberschleissheim, Germany. 31 pigs (15 control pigs and 16 pigs with ischaemic heart failure) were included in our analysis. Control pigs were matched to IHF pigs according to age and weight and underwent the same procedures as IHF pigs at day 30. Fig 1 shows a time-line depicting the procedures at every time point for each group as well as the number of animals.

Anaesthesia was induced by intramuscular application of ketamine (20 mg/kg), azaperone (10 mg/kg) and atropine (0,05 mg/kg) and was maintained after oral intubation with intravenous midazolam (0,5 mg/kg), fentanyl (0,05 mg/kg) and propofol (0,5 mg/kg/min). Pigs were mechanically and pressure-controlled ventilated (initial parameters: peak pressure 18–25 mmHg, peep 5 mmHg, tidal volume 6–8 ml/kg, FiO2 21%). Arterial blood gas tests were regularly performed to adjust ventilation parameters and to monitor electrolyte levels (sodium, potassium) which were adjusted to normal ranges throughout the experiments.

The internal jugular vein and the carotid artery of the right side were prepared by surgery and sheaths with appropriate size (8-F arterial sheath Avanti+, Cordis, USA and 9-F venous sheath Avanti+, Cordis, USA) were inserted. Heparin 5000 IU IV was injected after cannulation and insertion of the venous sheath.

On day 0 pigs received 150 mg amiodarone IV prior to coronary angiography to prevent ventricular tachyarrhythmias after LAD occlusion.

After myocardial infarction sheaths were removed, the wound was closed in layers, pigs were administered 50 mg/kg of intramuscular cefotaxime or cefuroxime to prevent bacterial wound infections, were brought back to their animal stalls and kept there until the final experimentation thirty days later. Control pigs were studied only at one time point.

For perioperative analgesic medication fentanyl (0,05 mg/kg, IV) and for postoperative analgesic medication buprenorphine (6 μg/kg, IM, twice per day for 2–3 days) was used. Alternatively, buprenorphine (6 μg/kg, IM) which was administered at least 30 minutes before the end of the experiment followed by carprofen (1,4 mg/kg, IV, at the end of the experiment and 4 mg/kg, PO, once per day for 3 days) was used for postoperative analgesia in IHF pigs. On day thirty anaesthesia was induced again and sheaths were inserted at the opposite cervical site.

### Coronary angiography and induction of myocardial infarction

Angiography was performed with a 6-F angiographic catheter (JL4 Infinity Thrulumen, Cordis, USA). For X-Ray imaging we used a Ziehm Vision mobile C-Arm (Ziehm imaging, Germany). Selective ventricular myocardial infarction was induced by occluding the left anterior descending artery distal to the first diagonal branch with a PTCA balloon catheter (15 mm length/3.5 mm diameter, max. balloon pressure 12–14 mmHg) for 90 minutes.

### Left heart catheterization

**Fig 2:**
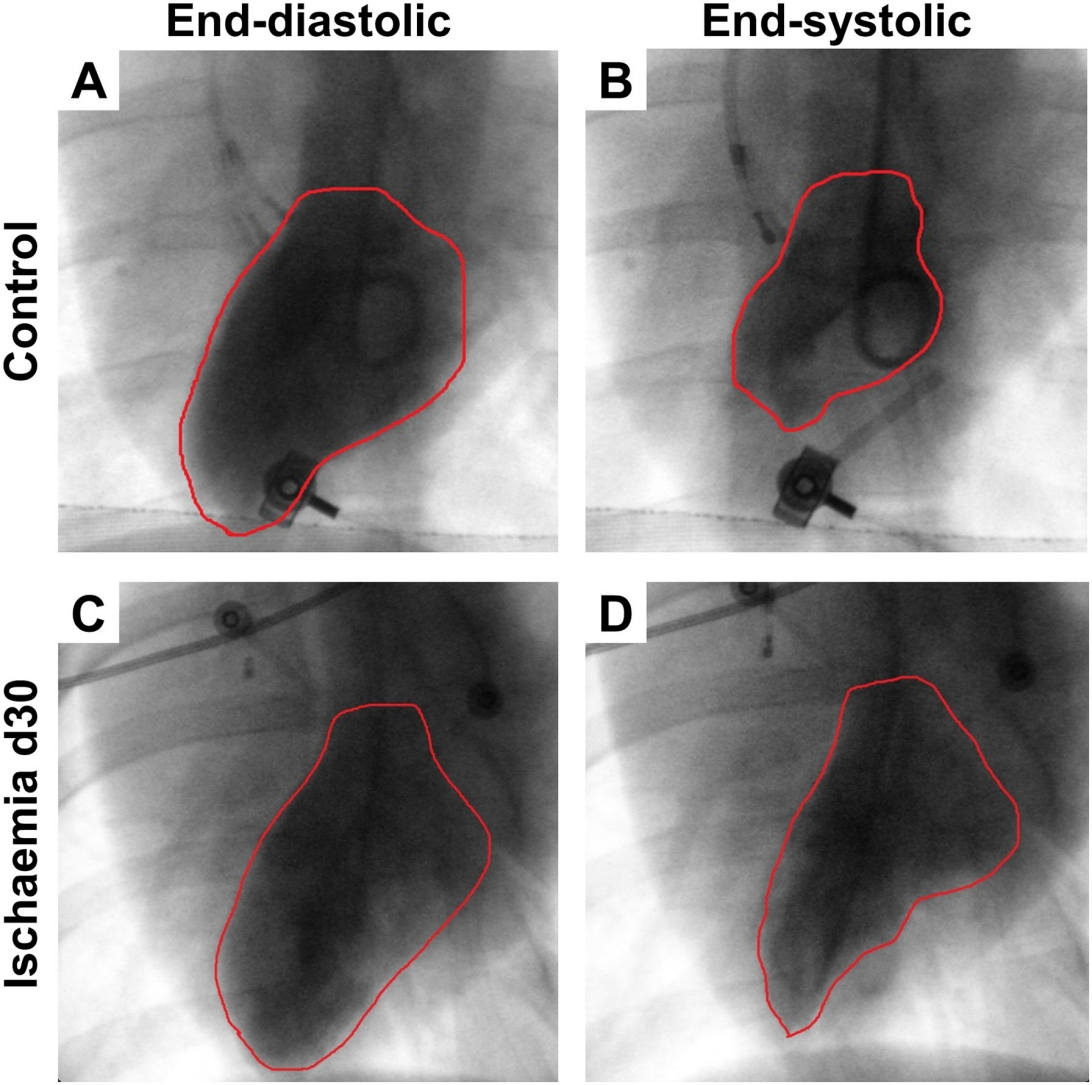
Representative levocardiography recordings. Representative levocardiography images of control pigs (A, B) and ischaemic pigs at day 30 (C, D). The area filled with contrast agent represents the end-diastolic (A, C) and end-systolic (B, D) left ventricular area and is highlighted by red lines.

To assess left ventricular ejection fraction, left ventricular levocardiography (6-F Super Torque Plus pigtail diagnostic catheter, Cordis, USA) was performed and recorded in two planes (anterior-posterior and 30° LAO projection) and afterwards quantified by a blinded investigator who was not involved in the operational procedures to avoid bias. Fig 2 provides representative images of levocardiography in systole and diastole of a control pig and an IHF pig. Left ventricular pressure (LVP) and left ventricular enddiastolic pressure (LVEDP) were invasively measured and monitored. Both LV pressure measurements and LV levocardiography were performed at a heart rate of 130 bpm to adjust for rate-dependent variations. For this purpose, a cardiac pacemaker lead (Edwards Lifesciences LLC, USA) was inserted into the right atrium via the venous sheath and connected to a pacemaker (Medtronic, Germany). Measurements were only performed in case of a 1:1 AV conduction.

### Right heart catheterization

Using a Swan-Ganz catheter (7-F Edwards Lifesciences LLC, USA) via the venous sheath we measured PA, RV, RA pressures and pulmonary capillary wedge pressure (PCWP).

### Electrocardiography (ECG)

12-lead ECGs were recorded prior to EP studies and P wave duration, PR interval and QRS duration were analysed by manually measuring 5 QRS complexes. Single-lead ECGs were recorded continuously throughout the experiment to monitor vital signs of each pig. Heart rate and QT/QTc duration were measured in single-lead ECGs 25 minutes after anaesthesia induction during steady-state anaesthesia. QT correction was performed with Bazett’s formula.

### Electrophysiological studies (EPS)

Electrophysiological studies of the right atria were performed with a MLCL CardioLab System (GE Healthcare, USA). A multipolar electrophysiology catheter was inserted via the venous sheath and placed near the sinus node at the high right atrium (HRA). All of the electrophysiological stimulations were executed in the high right atrium. The intrinsic basic cycle length (BCL) was determined by measuring the time period between two intrinsic atrial signals obtained with the EP catheter. Then, atrial pacing threshold was determined by pacing the atrium with a cycle length 20 ms shorter than the BCL and stepwise decreasing electrical output until the pacing is no longer effective. The following stimulation manoeuvres were then performed at 2x pacing threshold. For measurement of sinus node recovery time (SNRT) the atrium was paced with cycle lengths of 500 ms, 450 ms, 400 ms, 350 ms, 300 ms and 250 ms (twice each) for 30 seconds. SNRT was then measured as time period between the last stimulus and the first intrinsic atrial signal. Then, we continuously stimulated the atrium with a step-wise decreased cycle length until the induced atrial signal was no longer conducted 1:1 via the AV node (Wenckebach point) or was conducted in a 2:1 fashion (2:1 conduction cycle length). To assess effective refractory periods of the atrium and the AV node (AERP and AVERP) we stimulated the atrium with a train of eight stimuli at a fixed pacing cycle length followed by a premature stimulus at a continuously decreasing pacing cycle length until this premature stimulation was no longer conducted to the ventricles via the AV node (for AVERP) and did no longer induce an atrial signal (for AERP). This was performed at the following fixed pacing cycle lengths: 500 ms, 450 ms, 400 ms, 350 ms and 300 ms. Finally, AF was induced by rapid burst pacing at 1200 bpm (20 Hz) for 6 seconds. If no AF was induced, we waited for 30 seconds until we applied the next burst. In total, we delivered 10 bursts per pig. AF was defined as atrial arrhythmic episode with irregular RR intervals longer than 1 second. No IV antiarrhythmic substances or beta blockers were administered before EPS.

### Histological analysis and fibrosis quantification

**Fig 3:**
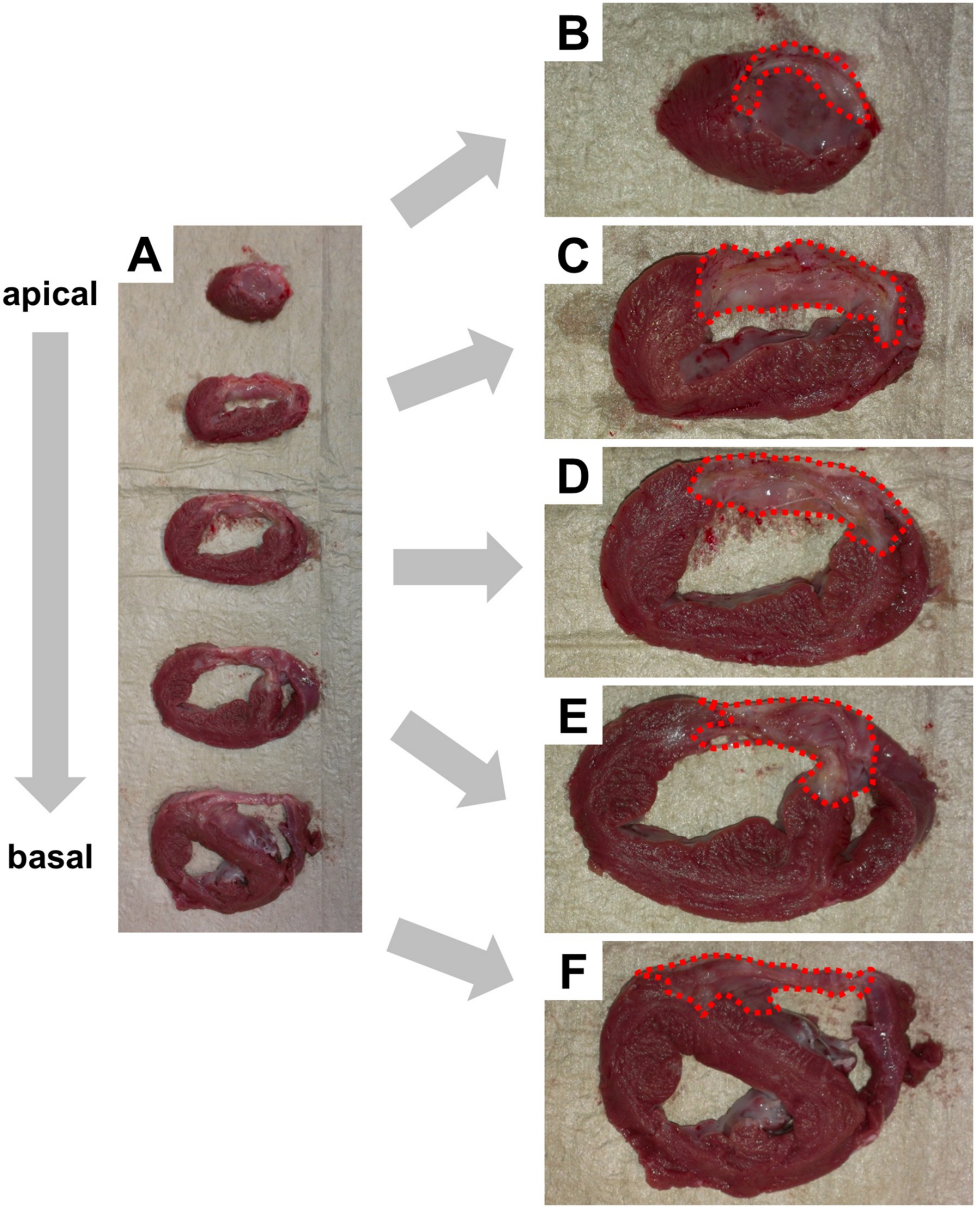
Gross anatomy. Hearts were cut into slices of 1 cm thickness from apex to basis (A). Representative images of an infarcted heart (B-F) illustrating scar size and the degree of left ventricular, septal and right ventricular infarction. Borders of infarcted areas are marked in red.

30 days after myocardial infarction hearts were removed in deep sedation (fentanyl 0,05 mg/kg, midazolam 0,5 mg/kg, propofol 0,5 mg/kg/min. IV) and tissue was prepared for histological analysis. Fig 3 shows representative images of an infarcted heart 30 days after LAD occlusion. The heart was cut into slices of approximately 1 cm thickness to illustrate the scar size and to determine the degree of left ventricular, septal and right ventricular infarction. Before performing a Masson’s Trichrome staining for collagen fibres (Masson-Goldner-Trichrome Staining Kit 3459.1, Carl Roth GmbH + Co. KG, Germany), paraffin-embedded sections (5 μm) were deparaffinized and rehydrated through alcohol abs., 96% alcohol, 70% alcohol and xylene. Pictures were taken with high-power field magnification (Axio Imager M2, Zeiss; 40-fold) and 12 non-overlapping pictures per region and animal were quantified using Adobe Photoshop CS3 Extended (collagen: blue; nuclei: black; muscle, cytoplasm: red). Slides for counting interstitial fibrosis were analysed in a blinded fashion to avoid bias in data analysis.

### Statistical methods

Data are presented as MEAN±SEM. The standard error of the mean (SEM) was calculated by dividing the standard deviation by the square root of n. GraphPad Prism 5.01 was used for statistical analysis. Mann-Whitney test was used for 2-group comparisons. Wilcoxon matched pairs test was used to compare measurements before and after onset of ischaemic heart failure. Categorical variables were analyzed by Fisher’s exact test. A p-value of <0.05 was considered statistically significant.

## Results

### Induction of an ischaemic heart failure

In total, we instrumented 43 consecutive age- and body weight-matched pigs (*German Landrace*, 3–4 months old, 50–70 kg body weight). Ventricular myocardial infarction was induced by balloon occlusion of the left anterior descending artery (LAD) distal to the first diagonal branch at day 0 (d0). After 90 minutes the balloon was removed to allow reperfusion. Both occlusion and reperfusion of the LAD were verified by coronary angiography. Selective ventricular infarction without atrial involvement was verified macroscopically after 30 days of reperfusion when the hearts were removed (d30). 6 pigs died during balloon occlusion due to ventricular fibrillation and 6 pigs died within 30 days of reperfusion due to heart failure related complications (sudden cardiac death (SCD)) and were therefore not included in our analysis. In total, we analyzed 31 pigs (16 pigs with ischaemic heart failure and 15 control pigs). Body weight (57.6±5.3 kg vs. 60.8±5.7 kg; p = 0.921) and heart/body weight ratio (4.2±0.08% vs. 3.9±0.07%; p = 0.07) did not significantly differ between control pigs and pigs 30 days after infarction.

### Haemodynamic characterization

**Fig 4:**
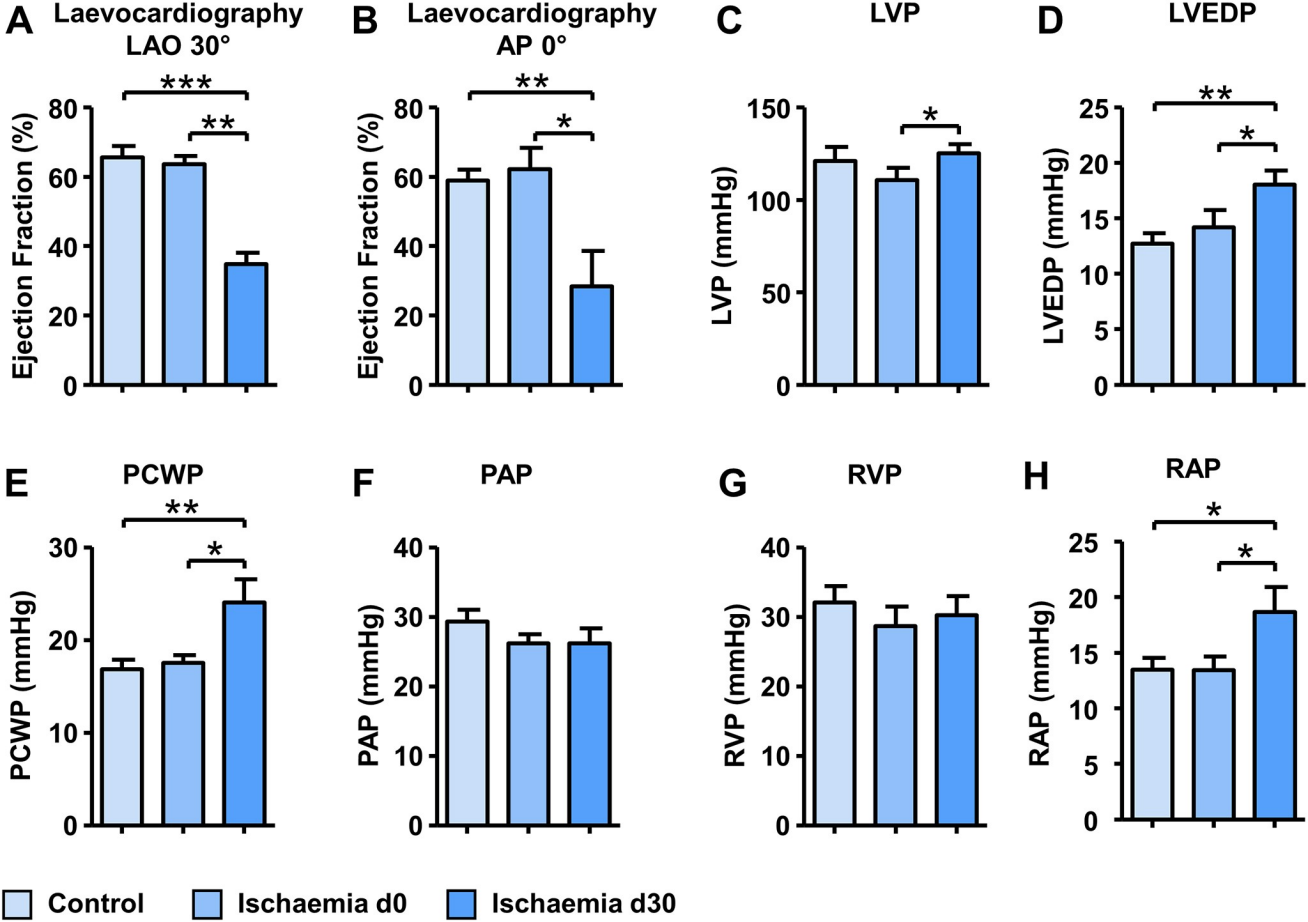
Haemodynamic measurements. Measurements of left ventricular ejection fraction by laevocardiography at LAO 30° (A) or AP 0° (B). Left ventricular (C), left ventricular end-diastolic pressure (D), pulmonary capillary wedge pressure (E), pulmonary artery pressure (F), right ventricular pressure (G) and right atrial pressure measurements (H) obtained via right heart catheterization in control pigs and ischaemic pigs at day 0 and day 30. LAO, left anterior oblique; AP, anterior-posterior; LVP, left ventricular pressure; LVEDP, left ventricular enddiastolic pressure; PCWP, pulmonary capillary wedge pressure; PAP, pulmonary artery pressure; RVP, right ventricular pressure; RAP, right atrial pressure.* p<0.05, ** p<0.01, *** p<0.001.

30 days after myocardial infarction pigs showed a markedly reduced ejection fraction both compared to baseline at d0 (63.5±2.4% vs. 34.8±3.3%; p = 0.002; Fig 4A and 63.2±2.1% vs. 29.2±3.6%; p = 0.031; Fig 4B) and control pigs (65.6±3.2% vs. 34.8±3.3%; p<0.0001; Fig 4A and 59.5±1.1% vs. 29.2±3.6%; p = 0.003; Fig 4B) demonstrating an ischaemic heart failure (IHF) phenotype. Representative images of levocardiography show reduced LV function in IHF pigs (Fig 2). Accordingly, haemodynamic parameters confirmed this distinct IHF phenotype: Left ventricular enddiastolic pressure (LVEDP) was elevated compared to baseline (d0) (14.2±1.5 mmHg vs. 18.0±1.3 mmHg, p = 0.038 Fig 4D) as well as compared to controls (12.7±0.9 mmHg vs. 18.0±1.3 mmHg, p = 0.005; Fig 4D). Furthermore, we observed increased pulmonary capillary wedge pressure (PCWP) and right atrial pressure (RAP) both compared to baseline (17.6±0.8 mmHg vs. 24.1±2.5 mmHg, p = 0.011 and 13.5±1.2 mmHg vs. 18.7±2.2 mmHg, p = 0.042; Fig 4E and 4F, respectively) and compared to controls (16.8±1.1 mmHg vs. 24.1±2.5 mmHg, p = 0.0047 and 13.5±1.0 mmHg vs. 18.7±2.2 mmHg, p = 0.020; Fig 4E and 4F, respectively). Left ventricular pressure (LVP), pulmonary artery pressure (PAP) and right ventricular pressure (RVP) did not differ between control and IHF pigs (Fig 4C, 4F and 4G).

### Electrophysiological characterization

To assess fundamental electrophysiological properties we obtained ECGs and performed invasive electrophysiology (EP) studies in controls and in pigs with IHF at d30.

Baseline ECG parameters such as heart rate, P wave duration, PR interval, QRS duration, and QTc interval did not differ significantly between groups (Table 1).

**Table 1:** ECG.

Parameter	Control	Ischaemia d30	p value
Heart Rate (bpm)^1^	79.9±3.4	74.7±3.8	0.402
P Wave Duration (ms)^2^	60.9±2.6	64.0±3.3	0.826
PR Interval (ms) ^2^	112.7±3.9	118.1±5.3	0.356
QRS duration (ms) ^2^	65.3±3.2	67.5±2.8	0.613
QTc Interval (ms) ^1^	374.5±10.6	370.1±12.0	0.718

bpm, beats per minute; ms, milliseconds.

^1^ measured by single lead ECG during steady-state.

^2^ measured by 12-lead ECG.

During electrophysiology studies we applied standardized programmed stimulation protocols to measure electrophysiological properties of the sinus node, the atrium, and the AV node. Relative sinus node recovery time (SNRT/BCL) at three different cycle lengths did not significantly differ between groups (Table 2). Atrial effective refractory periods (AERP) were shorter in IHF pigs compared to controls at all five different basic cycle lengths measured but did not reach statistical significance. AV nodal electrical properties measured by Wenckebach cycle length and AV nodal effective refractory periods (AVERP) did also not differ significantly between groups (Table 2).

**Table 2:** Electrophysiological properties.

Parameter	Control	Ischaemia d30	p value
Sinus Node Recovery Time (SNRT)			
SNRT_500ms_/BCL (%)	114.8±9.3	151.0±13.3	0.100
SNRT_450ms_/BCL (%)	121.0±5.3	163.8±15.6	0.076
SNRT_400ms_/BCL (%)	126.3±8.4	167.9±20.5	0.330
Atrial Effective Refractory Period (AERP)			
AERP_500ms_ (ms)	230.8±16.3	206.0±15.0	0.293
AERP_450ms_ (ms)	223.6±13.2	208.1±13.1	0.392
AERP_400ms_ (ms)	216.4±12.0	201.9±11.5	0.348
AERP_350ms_ (ms)	207.9±12.1	190.0±10.2	0.380
AERP_300ms_ (ms)	200.8±12.3	190.0±11.8	0.535
AV Wenckebach Cycle Length (ms)	220.0±9.5	245.6±10.2	0.123
AV Node Effective Refractory Period (AVERP)			
AVERP_500ms_ (ms)	246.7±14.5	260.0±11.4	0.576
AVERP_450ms_ (ms)	226.0±15.0	261.1±10.7	0.094
AVERP_400ms_ (ms)	216.0±20.1	240.0±11.8	0.281
AVERP_350ms_ (ms)	206.0±20.6	216.7±10.9	0.687
AVERP_300ms_ (ms)	188.0±8.6	187.1±12.5	1.000

ms, milliseconds; BCL, basic cycle length.

### Atrial arrhythmogenicity

**Fig 5:**
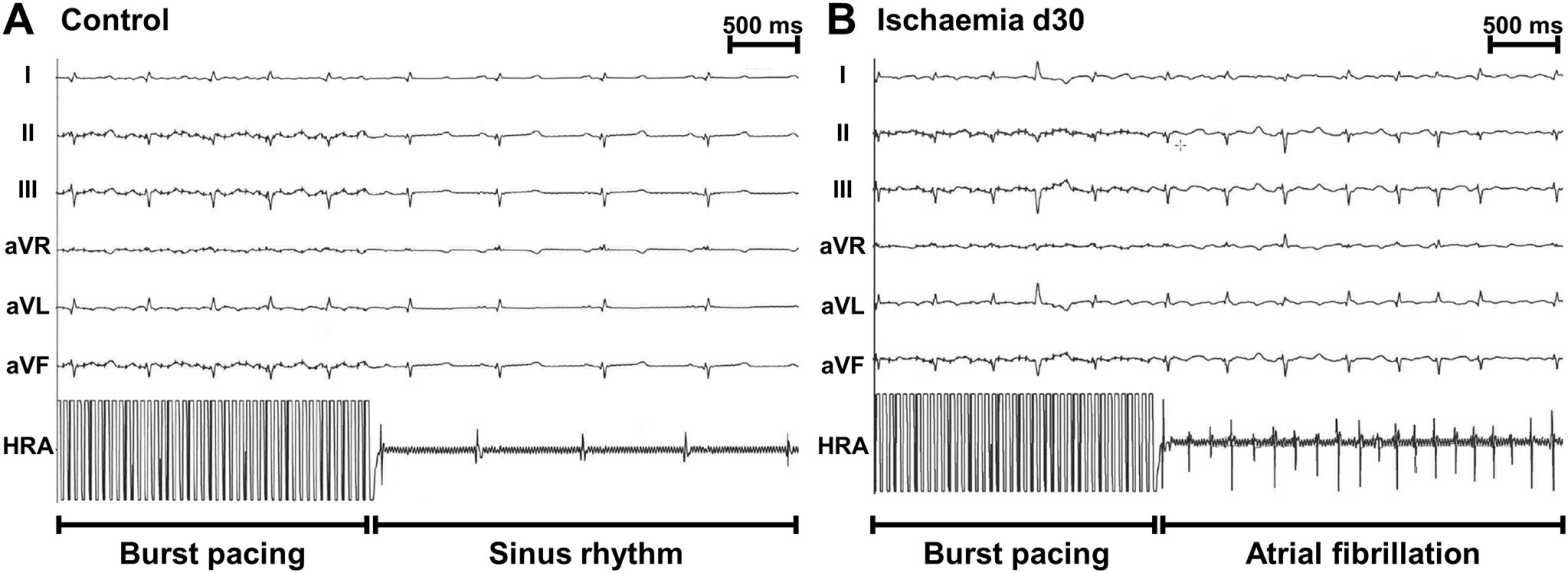
Representative ECG tracings. Representative ECG tracings obtained during EP studies in a control pig (A) and an ischaemic pig at day 30 (B) illustrating atrial burst pacing at 1200 bpm. The upper tracings represent the surface ECG leads I, II, III, aVR, aVL, aVF, the tracing at the bottom represents the intracardiac electrogram recorded at the tip of the EP catheter placed in high right atrium (HRA). Tracings were recorded at 50 mm/s.

Following electrophysiology studies we performed right atrial burst pacing at 1200 bpm to assess the susceptibility to atrial arrhythmias. Fig 5 shows representative ECG tracings illustrating the onset of AF after burst pacing in an ischaemic pig whereas in a control pig burst pacing was not successful to induce AF.

**Fig 6:**
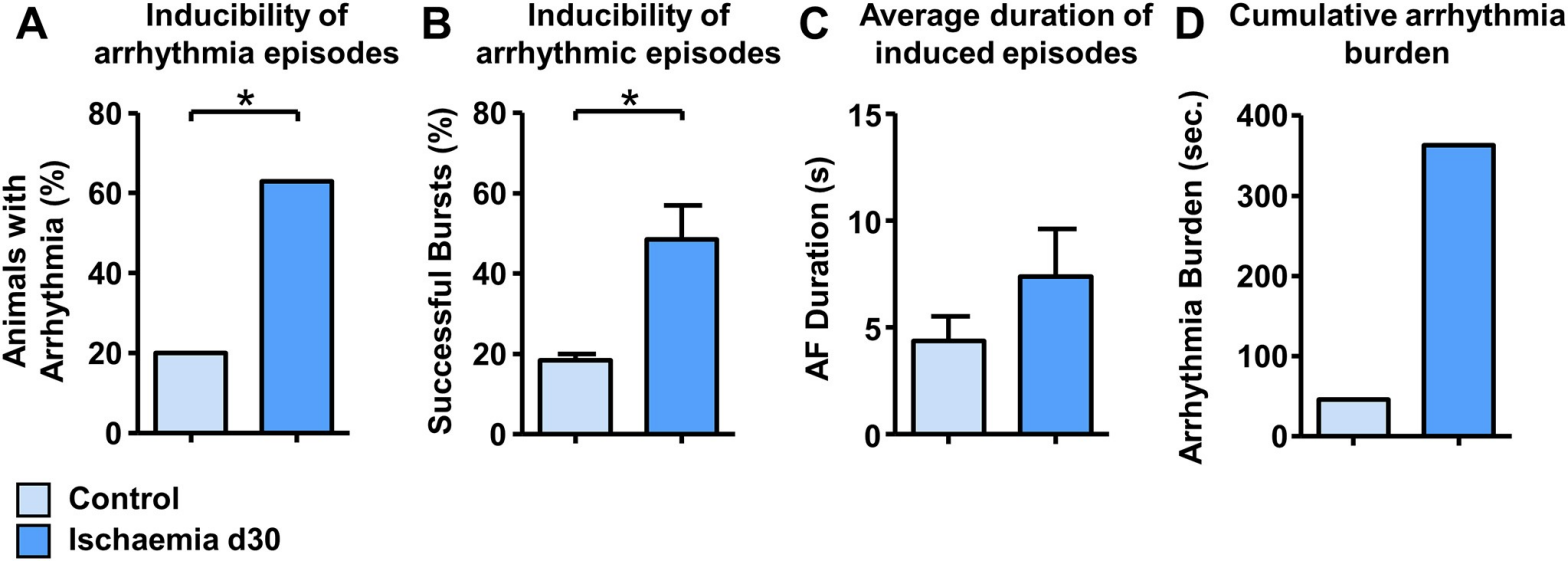
Electrophysiology study. Inducibility of atrial arrhythmia episodes per animal (A), percentage of successful bursts (B), average duration of induced arrhythmia episodes (C), and cumulative arrhythmia burden (D) in control and ischaemic pigs at day 30.* p<0.05, ** p<0.01, *** p<0.001.

In pigs with IHF we observed a significantly increased atrial arrhythmogeneity. IHF pigs demonstrated a significantly higher inducibility of AF compared to control pigs (3/15 vs. 10/16 pigs, p = 0.029; Fig 6A) and in IHF pigs a significantly higher number of bursts lead to AF episodes compared to controls (18.33±1.7% vs. 48.5±8.4%, p = 0.046 Fig 6B). Furthermore, the average duration of induced arrhythmia episodes was longer in IHF pigs (4.37±1.2 sec. vs. 7.4±2.2 sec., Fig 6C) and the AF burden demonstrated as cumulative duration of all induced AF episodes was markedly increased in IHF pigs (46.1 sec vs. 363.2 sec., Fig 6D) without reaching statistical significance.

### Histological analysis

Since structural remodeling is one of the hallmarks of AF pathophysiology we assessed the percentage of interstitial fibrosis in formalin-fixed, paraffin embedded tissue stained with Masson’s trichrome.

**Fig 7:**
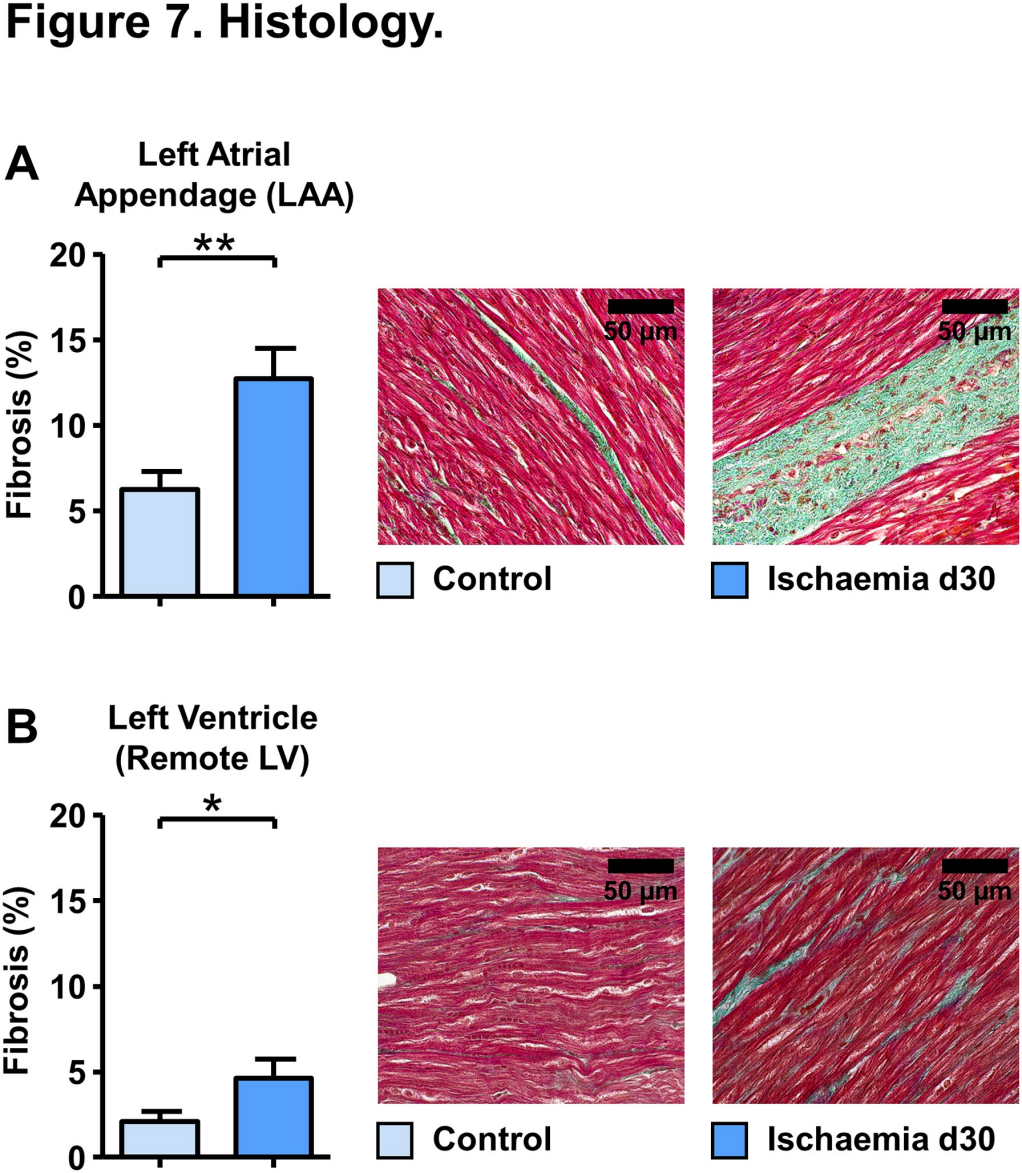
Histology. Quantification of fibrosis in left atrial appendages (A) and remote left ventricle (B) between control pigs and ischaemic pigs at day 30 after AMI. * p<0.05, ** p<0.01.

Both in left atrial appendages (LAA) and left ventricular (LV) tissue (remote of infarction) we observed significantly increased amounts of fibrosis in IHF pigs 30 days after myocardial infarction compared to controls (6.3% vs. 12.8%, p = 0.008; Fig 7A; and 2.1% vs. 4.6%, p = 0.042; Fig 7B, respectively) potentially demonstrating a proarrhythmic structural substrate.

## Discussion

Here, we were able to identify electrophysiological alterations in a porcine model of ischaemia-reperfusion injury which closely resembles the alterations after myocardial infarction found in patients, including haemodynamic, functional or structural changes essentially leading to enhanced atrial arrhythmogenicity. This model is therefore clinically highly relevant and offers the opportunity to study especially early mechanisms that underly atrial arrhythmogenicity in the context of IHF.

The development of suitable large animal models is highly necessary in order to investigate pathomechanisms of arrhythmogenesis after AMI, to translate basic science findings into clinical practice and to test novel treatment approaches. Clearly, rodents and other small animal models serve as vital models in basic research and provide advantages such as standardized genetic backgrounds, transgenic strains, wide availability, reasonable housing costs, ease of maintenance and short gestation times. Nevertheless small animal models do have several limitations especially in comparison to large animal models such as the pig. Due to the small heart size there is not enough tissue to allow gene/protein expression studies, histology and cell isolation from the same animal and several measurements are not possible at all or only to a limited degree such as coronary angiography, haemodynamic measurements or electrophysiology studies. Furthermore, the rodent electrophysiology is quite different compared to humans in regard to ion channel expression and distribution, action potential shape and duration, repolarization, or heart rate, and thus, limits the use of rodent models for translational studies [7]. Pigs could overcome these limitations since their cardiac and coronary anatomy, haemodynamics or electrophysiology are similar to humans, all measurements performed in patients can also be done in pigs, and there is enough tissue available for comprehensive, chamber- and even region-specific studies. A porcine model of IHF could therefore be helpful to further investigate underlying mechanisms of atrial arrhythmogenicity including structural, electrical and autonomic remodeling as well as inflammatory or neurohumoral changes.

Various large animal models have been investigated so far to study the pathophysiology of AF [7]. As pathophysiology of AF remains complex, different models with various disease triggers and with focus on different aspects of AF have been developed. The majority of AF models have been using tachypacing with custom built pacemakers to maintain and generate a substrate for AF starting with the pioneering experiments by Wijffels et al. in 1995 who demonstrated that tachycardia-induced remodeling is an underlying mechanism for AF [11]. So far, tachypacing models have been applied in goats, sheep, rabbits, dogs and pigs and have been demonstrated to cause AF [7]. Nevertheless, these atrial tachypacing models have limitations such as the necessity to slow down AV conduction interventionally or medically in order to prevent onset of tachymyopathy as potential confounding trigger. Ventricular tachypacing, on the other hand, causes tachycardiomyopathy with secondary atrial remodeling. Although it shows severe heart failure, mimics aspects seen in dilative cardiomyopathy, and has provided highly valuable insights into mechanisms leading to arrhythmias, it models a phenotype that is less prominent than ischaemic heart failure in clinical routine. In these models tachymyopathy causes AF while in the clinical setting AF with fast AV nodal conduction (as a consequence of insufficient rate control) may contribute to heart failure as a form of tachymyopathy [12]. Therefore these models do not perfectly resemble common clinical situations and thus, any conclusions drawn from these models regarding clinical treatment do probably not apply for patients with a more common cause of AF such as ischaemic heart failure.

As ischaemic cardiomyopathy is a major cause for the development of AF and myocardial infarction is an independent risk factor for AF [8] a large animal model of myocardial ischaemia/ischaemic heart failure could potentially represent a highly useful model to study AF pathophysiology. In mouse and rat models fundamental ischaemia-related mechanisms of arrhythmogenesis can be studied but translating those findings into clinical practice requires validation and preclinical testing in large animals. For this reason, both ventricular and atrial infarction have been applied to dogs in the past demonstrating increased occurrence of AF in these models [7]. However, translating these findings to human pathophysiology is difficult due to various reasons: dogs have an extensive coronary collateralization and the induction of sufficient ischaemia is challenging [7]. Despite these problems canine atrial as well as ventricular ischaemia models have been successfully induced and occurrence of AF has been studied in these models [13, 14], but ethical considerations and lower social acceptance limit the use of dogs in many countries raising the need for other large animal models. Pigs could serve as ideal species since the porcine cardiac anatomy, electrophysiology and coronary circulation is highly similar to those of humans, reproducible infarct sizes can be easily achieved, they are widely available at reasonable costs, and the acceptance by societies is higher compared to dogs [7]. Therefore, to avoid these limitations of dog models, our aim was to investigate a pig model reliably mimicking the human phenotype of ischaemic heart failure in order to study ischaemia-induced atrial remodeling and its proarrhythmic atrial substrate.

In the present study selective ventricular infarction via transient occlusion of the LAD distal to the first diagonal branch caused a clear phenotype of IHF 30 days after infarction. This was detectable both compared to control pigs and to pigs before infarction: Left ventricular catheterization and levocardiography demonstrated markedly reduced left ventricular EF as well as elevated LVEDP. Right heart catheterization showed significantly elevated capillary wedge pressures and right atrial pressures, whereas pulmonary artery and right ventricular pressures remained unchanged. As myocardial infarction leads to heart failure, LVP usually decreases due to reduced contractile capability of the myocardium. In our study we observed no statistical difference in peak systolic LVP comparing the group of age- and weight-matched control pigs with IHF pigs (Ischaemia d30). The significant increase in LVP between pigs prior and post infarction (Ischaemia d0 vs. Ischaemia d30) might be due to an age-related effect since pigs at this age are rapidly growing and gaining weight. To proof this hypothesis it is necessary to–according to the ischaemia group–study control pigs at two different time points, but unfortunately, this was not allowed by the local ethics committee. Elevated right atrial pressures might be due to a decreased right ventricular function following AMI especially as macroscopic analysis of infarcted hearts showed involvement of the RV septum and RV apex. As we did not observe any papillary muscle involvement or macroscopic destruction of tricuspid valves in none of the animals we assume that the predominant reason for the observed elevated RA pressure is RV dysfunction. Furthermore, IHF pigs were more susceptible for atrial arrhythmic episodes, a higher percentage of stimulation manoeuvres led to those episodes and induced episodes were longer. Since sustained episodes were not observed, IHF pigs develop a phenotype that might be comparable to early phases of human paroxysmal AF after AMI with short and self-limiting atrial arrhythmic episodes.

In patients with ischaemic heart failure morphologic changes such as LA dilatation can be typically observed [9]. These atrial alterations occur secondary due to deranged haemodynamics in ischaemic ventricles independent of atrial ischaemia. Due to these effects on the atria, ischaemic heart failure is highly associated with AF [15], a finding that we could confirm in our IHF pigs. Although there are multiple triggers for arrhythmogenic remodeling [3], altered haemodynamics with increased atrial stretch have been recently demonstrated as important factors since ventricular unloading after AMI with an Impella system has been shown to successfully reduce LA stretch and AF in pigs [16].

Besides its effect on atrial stretch, ventricular ischaemia can also affect electrophysiological properties (e.g. atrial refractory periods) of the atria [17]. These electrophysiological alterations observed in patients have been confirmed in animal models, mainly in dogs after atrial infarction [13]. Selective ventricular infarction, however, does not necessarily affect conduction velocity or refractory periods as demonstrated in rabbits [18]. In pigs, only few groups measured ventricular refractory periods after infarction, data on atrial effective refractory periods in the context of IHF, however, are not available so far.

Electrophysiological properties such as ECG parameters or atrial refractory periods were unchanged among groups. Thus, we focused on investigating structural rather than electrical remodeling in our study and evaluated fibrosis as the hallmark in structural remodeling. Atrial fibrosis through accumulation of collagen fibres usually occurs in reparative processes to replace degenerated cardiomyocytes [19] with atria being more sensitive for fibrosis than ventricles in heart failure [20]. Heart failure with haemodynamic overload enhances atrial dilatation and stimulates profibrotic signalling pathways, e.g. the TGF-β pathway [19]. Fibrosis sets basis for conduction heterogeneity of the atria leading to vulnerability for AF occurrence. The association between atrial fibrosis and AF has been highlighted in many studies so far and fibrosis correlates with AF occurrence and persistence [21]. Our histological analyses revealed increased fibrosis in the left atrium as a structural correlate of conduction heterogeneity most likely to facilitate arrhythmia generation.

As we found no overt electrical abnormalities, structural remodeling due to altered haemodynamics with increased atrial pressure leading to enhanced atrial fibrosis seems to be a key player in generating an early arrhythmogenic atrial substrate for AF in IHF pigs 30 day after selective ventricular myocardial infarction.

### Study limitations

We evaluated the changes in haemodynamics, atrial structure and arrhythmogenicity 30 days after myocardial infarction in pigs. We induced AMI by balloon occlusion of the LAD for 90 minutes. However, AMI in humans is most commonly caused by atherosclerotic plaque rupture with subsequent occlusion of the vessel by an immunothrombosis. Nevertheless, modern and fast revascularization techniques ensure reperfusion of coronary vessels within short time which we mimic in our model (reperfusion after 90 minutes). Since pigs have a faster infarct progression compared to humans [22], they might not be perfect to investigate acute effects of AMI but they allow to investigate effects of IHF already after 30 days. As this pig model shows haemodynamic changes similar to those observed in patients after AMI, it seems to be highly suitable to study IHF and its secondary effects, e.g. on arrhythmogenesis. However, as mentioned above, this model might not be suitable to study the causes of myocardial ischaemia such as atherosclerosis. The idea of a porcine model of IHF is not completely new: Ischaemia-reperfusion has been performed in various animal models including pigs to study various IHF-induced alterations, in most cases in regard to scar formation, LV function, or heart failure [7]. Ishikawa et al. investigated ventricular unloading in IHF pigs and were the first to report the occurrence of AF in this model (which is reduced after ventricular unloading), but did not perform standardized *in vivo* electrophysiology studies to obtain electrophysiologic parameters in this model [16]. Our study adds a comprehensive electrophysiologic characterization of the atrium, but interestingly, we could not observe any overt alterations in electrical properties *in vivo*. On the other hand, we could confirm that ventricular infarction leads to enhanced atrial arrhythmogenicity as observed in other species suggesting that mechanisms other than altered electrical properties are the main contributors to arrhythmogenesis in this model.

As mentioned above, we focused on the inducibility of atrial arrhythmic episodes and observed only short and self-limiting episodes. Also, as we did not use implantable loop recorders throughout the 30 days after AMI we are not able to report any spontaneous arrhythmia episodes. Thus, we cannot call this model a ‘paroxysmal AF model’, but clearly demonstrated a higher susceptibility to short and non-sustained atrial arrhythmias. Therefore, this model might resemble a very early phase of proarrhythmic remodeling establishing a substrate sufficient to make the heart vulnerable for the onset of arrhythmias, but not yet sufficient enough to maintain an arrhythmic episode. Thus, the model we describe here might be highly valuable to study early proarrhythmic remodeling especially in terms of prevention. Nevertheless, more studies are warranted to investigate for example the potential occurrence of spontaneous arrhythmic episodes via implantable loop recorders.

Our study suggests that structural changes after myocardial infarction might be a potential underlying mechanism of the enhanced atrial arrhythmogenicity we observed. However, based on our current data we are not able to provide clear evidence of the causal link between structural remodeling and enhanced arrhythmogenicity. This has to be investigated in future studies. Furthermore, as we focused on atrial electrophysiological processes we did not record dP/dtmax and dP/dtmin and did not perform volume measurements of heart chambers which might have given more detailed information about haemodynamic alterations and especially ventricular remodeling processes.

As 150 mg of amiodarone was administered before myocardial infarction in order to reduce the number of VF during PTCA balloon occlusion it might restrain the occurrence of AF 30 days after AMI due to its long elimination half-life. This might have attenuated the effects seen in our study. Nevertheless it most likely reduced the number of VF-related deaths during AMI.

We compared pigs with IHF to age- and weight-matched control pigs without prior instrumentation. Although true sham controls, i.e. pigs with coronary angiography but without balloon occlusion and a second evaluation 30 days later would be even more suitable controls, we think that our approach was justified especially in terms of animal welfare.

## Conclusions

Selective ventricular infarction in pigs causes ischaemic heart failure with elevated atrial pressure leading to increased left atrial fibrosis generating an arrhythmogenic substrate. We demonstrate that a porcine model of ischaemic heart failure reliably resembles the human situation after myocardial infarction and could therefore serve as valuable model to further investigate underlying mechanisms of atrial arrhythmogenicity in ischaemic heart failure. This preclinical model could be vital especially for translational studies to validate and test potential drug targets identified in rodents and to develop novel and innovative therapeutic approaches in a close-to-human large animal model.

## Supporting information

S1 File (XLSX)
